# Altered Risk-Taking Behavior in Early-Stage Bipolar Disorder With a History of Psychosis

**DOI:** 10.3389/fpsyt.2021.763545

**Published:** 2021-11-18

**Authors:** Sandra Chi Yiu Wong, Mary Chung Mun Ng, Joe Kwun Nam Chan, Martha Sin Ki Luk, Simon Sai Yu Lui, Eric Yu Hai Chen, Wing Chung Chang

**Affiliations:** ^1^Department of Psychiatry, Queen Mary Hospital, The University of Hong Kong, Pokfulam, Hong Kong SAR, China; ^2^State Key Laboratory of Brain and Cognitive Sciences, The University of Hong Kong, Pokfulam, Hong Kong SAR, China

**Keywords:** risk taking, risky decision-making, bipolar disorder, psychosis, BART 2

## Abstract

Altered risk-taking propensity is an important determinant of functional impairment in bipolar disorder. However, prior studies primarily assessed patients with chronic illness, and risk-taking has not been evaluated in the early illness course. This study investigated risk-taking behavior in 39 euthymic early-stage bipolar disorder patients aged 16–40 years who were treated within 3 years from their first-episode mania with psychotic features and 36 demographically-matched healthy controls using the Balloon Analog Risk Task (BART), a well-validated risk-taking performance-based paradigm requiring participants to make responses for cumulative gain at increasing risk of loss. Relationships of risk-taking indices with symptoms, self-reported impulsivity, cognitive functions, and treatment characteristics were also assessed. Our results showed that patients exhibited significantly lower adjusted scores (i.e., average balloon pumps in unexploded trials) (*p* = 0.001), lower explosion rate (*p* = 0.007) and lower cumulative scores (*p* = 0.003) than controls on BART, indicating their suboptimal risk-taking performance with increased propensity for risk aversion. Risk-taking indices were not correlated with any symptom dimensions, self-reported impulsivity, cognitive functions or antipsychotic dose. No significant difference was observed between patients with and without antipsychotic medications on self-reported impulsivity or any of the BART performance indices. This is the first study to examine risk-taking behavior in early-stage bipolar disorder with history of psychosis and indicates that patients displayed altered risk-taking with increased risk aversion compared with controls. Further research is needed to clarify longitudinal trajectory of risk-taking propensity and its relationships with psychosis and functional outcome in the early stage of bipolar disorder.

## Introduction

Heightened risk-taking has been regarded as an important clinical feature of bipolar disorder and is associated with maladaptive behaviors such as substance abuse, and functional impairment. A growing body of studies have recently been conducted to better characterize risk-taking propensity in bipolar disorder using performance-based measures. Earlier research mainly applied Iowa gambling task (IGT) (1) as a behavioral index of risk-taking and generally showed that patients with bipolar disorder displayed impairment in IGT performance, with increased preference for the disadvantageous decks (i.e., risky choices) (2). However, accumulating data indicated that deficient IGT performance might primarily stem from impairment in trial-by-trial estimation of expected value in associative learning and working memory dysfunction (3–5). A recent meta-analysis (2), which included six prior studies that formally investigated risk-taking propensity in bipolar disorder based on performance in the Balloon Analog Risk task (BART) (6–8) or the Cambridge Gambling task (CGT) (9–11), revealed lack of overall significant impairment in risk-taking behavior in patients relative to healthy controls. Notably, discrepant findings were observed across individual studies. For instance, among those three reports examining the BART in bipolar disorder, one demonstrated increased risk-taking in patients (7), another found elevated risk-taking only in patients with comorbid alcohol dependence (6), while the remaining study noted increased risk aversion among patients taking antipsychotic medications compared to those without antipsychotic treatment (8).

In fact, such mixed findings may partly be attributable to the clinical heterogeneity among study samples. In these studies, recruited patients were mixed in varying proportions of different mood states (euthymia, mania or depression), disorder subtypes (type I or II), history of psychosis, and concurrent substance or alcohol abuse (2). Many studies also did not report information regarding medication treatment, especially the use of antipsychotics. All of the past studies evaluating risk-taking behavior in bipolar disorder focused on patients with chronic illness. Until now, there has been no published report examining risk-taking behaviors using performance-based assessment in the early-stage bipolar disorder. This is, however, of significant clinical implications. In particular, evidence has consistently shown that patients frequently experience persistent functional disability even after achieving clinical remission from their first-episode mania (12). Previous studies have further suggested that bipolar disorder patients with history of psychosis exhibit worse clinical and functional outcomes than those without history of psychosis. Thus, investigation and better characterization of risk-taking in patients with early-stage bipolar disorder with psychosis (BD-P) may facilitate development of effective interventions to promote early functional recovery.

Alternatively, substantial research has examined impulsivity in bipolar disorder, primarily using self-reported assessment, and generally found elevated levels of impulsivity in patients relative to healthy controls and across different affective states of the illness including euthymia (13, 14). Of note, impulsivity and risk-taking are related but separable constructs, with the former reflecting reduced control over behavior (in response to stimuli) (15, 16) while the latter referring to engaging in behavior with a high potential for harm and simultaneous opportunity for reward (assumed as a selected strategic response) (16, 17). Prior studies mostly revealed weak or lack of significant correlations between risk-taking behavioral measures and self-reported impulsivity in patients with chronic bipolar disorder (6, 8). Relationship between these *two* variables in the early stage of illness remains to be clarified.

In the current study, we sought to examine risk-taking propensity in a cohort of euthymic Chinese patients with early-stage BD-P, who had received <3 years of treatment following their first-episode mania with psychotic features, using the BART. Briefly, the BART is a well-established and ecologically-valid performance-based measure of risk-taking behavior, and participants are required to choose risky vs. safe options, with risk-taking being indexed as a tendency to withhold option selection until rewards of greater magnitude (but also associated with risk of corresponding greater penalties) are presented (18). This behavioral paradigm has previously been studied in our local Chinese chronic schizophrenia and early non-affective psychosis samples (19, 20). We also aimed to explore the relationships of risk-taking measures with clinical profiles, self-reported impulsivity traits, cognitive functions, and treatment characteristics in early-stage BD-P.

## Methods

### Participants and Study Setting

Thirty-nine patients aged 16–40 years with DSM-IV (21) diagnosis of bipolar I disorder were recruited from outpatient units of a territory-wide specialized early intervention service for psychosis (namely EASY programme) (22, 23) in Hong Kong within 3 years following treatment initiation for their first-episode mania with psychotic features. Diagnosis was ascertained by the Chinese-bilingual Structured Clinical Interview for DSM-IV (CB-SCID-I/P) (24) and medical record review. The clinically-stable BD-P patients were enrolled if they did not have recurrence of manic episode and fulfilled the following criteria for at least 2 months before study assessment: (1) had been in euthymic mood state as defined by an absence of a mood episode based on clinical interviews (according to CB-SCID-IV mood module criteria) as well as cutoff scores ≤ 7 on both the Young Mania Rating Scale (YMRS) (25) and Hamilton Rating Scale for Depression (HAM-D) (26); and (2) had been on stable medication regimen.

Thirty-six demographically-matched healthy controls were recruited from the community via advertisements and word-of-mouth among recruited participants. Controls with psychiatric diagnosis verified by CB-SCID-I/P, family history of mood or psychotic disorder, or were taking any psychotropic medications were excluded from the study. General exclusion criteria for all study participants were intellectual disability, neurological disease, history of head injury, or substance abuse in the past 6 months (assessed by the Alcohol Use Scale and the Drug Use Scale) (27). The study was approved by the local institutional review boards. Written informed consent was obtained from all participants, and parental consent was also sought for those aged under 18 years.

### Clinical and Cognitive Assessments

Current symptoms of mania and depression were measured using YMRS and HAM-D, respectively. Positive, disorganization and negative symptoms were evaluated by the Positive and Negative Syndrome Scale (PANSS) (28). Barratt Impulsiveness Scale-11 (BIS-11) Chinese version (29, 30) was employed to assess participants' impulsivity traits and comprised three separate dimensions including attentional, motor and non-planning impulsiveness. A brief battery of cognitive assessments was administered to all participants, including letter-number span (31), digit symbol coding subtest from the Wechsler Adult Intelligence Scale-Revised (32), letter cancellation test (33), and logical memory subtest from the Wechsler Memory Scale-Revised (34).

### Risk-Taking Behavioral Paradigm

A computerized risk-taking behavioral paradigm, namely the Balloon Analog Risk Task (BART) (18) was administered to each participant. Details of the BART has been described in our prior study (19) and the same task parameters were adopted in the current investigation. Briefly, participants were presented with a simulated balloon and a balloon pump on a computer screen per trial. Each key-press inflated the balloon slightly and one point was gained and deposited in a temporary pool. At any time before the balloon exploded, participants were given an option to inflate the balloon to attain more points or to stop pumping so as to collect and transfer the accrued points from the temporary pool to a permanent repository. Participants would lose all the accrued points for that trial if the balloon was exploded. Thus, a larger balloon indicated higher magnitude of reward as well as greater risk of loss by explosion. The task consisted of 20 trials. In each trial, the balloon was set randomly to explode between 1st and 128th pump. Participants were instructed to accrue as many points as possible in the permanent repository. Number of balloon pumps made per trial and cumulative score in the permanent repository were displayed on the screen. Three risk-taking indices were then derived from task performance. We first generated adjusted score which was defined as the average number of pumps for each trial with unexploded balloon, with higher score denoting greater risk-taking propensity. We also computed rate of exploded balloons and cumulative score (i.e., total points accrued in the permanent repository) for analyses.

### Statistical Analysis

Demographics, cognitive functions and self-reported impulsivity traits were compared between patients and controls using chi-squared test and independent-samples *t*-test, as appropriate. Group differences on three risk-taking indices of BART, namely adjusted score, exploration rate and cumulative score, were examined using independent-samples *t*-tests. Correlation analyses were conducted to assess the associations of BART performance measures with symptom dimensions, self-reported impulsivity traits, antipsychotic doses (as quantified by chlorpromazine equivalents) (35), individual cognitive test scores, and cognitive composite score which was calculated as a measure of general cognitive function by averaging standardized z-scores of individual cognitive tests, with z-score for each cognitive test being computed on the basis of controls' performance. As previous research suggested that bipolar disorder patients taking antipsychotics may be more risk-averse than those not taking antipsychotics (8), exploratory comparison analyses were conducted between patients with (*n* = 30, all on second-generation antipsychotics) versus without (*n* = 9) antipsychotic treatment in terms of demographics, clinical profiles, cognitive functions, impulsivity traits, and BART performance measures. Additionally, to explore potential effects of lithium and valproate treatment as *well* as past depressive episode on risk-taking measures, we performed the respective subgroup comparison analyses (*i.e*., on lithium *vs*. not on lithium; on valproate *vs*. not on valproate; and with *vs*. without past depressive episode) in patient characteristics and BART performance. Based on a recent study demonstrating significant difference between euthymic BD patients and healthy participants on BART performance (7), 20 patients and 20 controls would be required to detect significant group difference with 80% power at 0.05 significance level. The level of statistical significance for all analyses was set at *p* < 0.05.

## Results

### Characteristics of the Sample

Demographics, cognitive functions, clinical and treatment characteristics of the participants are summarized in Table 1. No significant differences were noted between patients and controls in age, gender or educational levels. Patients performed significantly worse in all individual cognitive tests than controls. The two groups did not differ in BIS-11 total, attentional and motor scores, but showed trend-wise significant difference in BIS-11 non-planning impulsiveness scores (with higher scores in patients; *p* = 0.06).

**Table 1:** Demographics, cognitive functions and clinical characteristics of patients and controls^a^.

	**Patients**	**Controls**	**Statistics^**b**^**	
**Variables^**a**^**	**(*n* = 39)**	**(*n* = 36)**	**(t/χ^2^)**	***p-*value**
**Demographics**				
Age in years	24.4 (6.2)	24.7 (7.0)	−0.2	0.85
Male gender, *n* (%)	16 (41.0)	17 (47.2)	0.8	0.59
Years of education	13.6 (2.8)	14.8 (2.9)	−1.7	0.09
**Cognitive functions**				
Letter-number span	14.3 (4.0)	17.3 (2.8)	−3.7	<0.001
Digit symbol coding	11.9 (3.3)	14.7 (2.8)	−4.0	<0.001
Logical memory	10.6 (4.5)	13.8 (3.8)	−3.4	0.001
Letter cancellation^c^	3.9 (3.7)	2.4 (2.8)	2.0	0.04
**Impulsivity trait measures**				
BIS-11 total score	67.7 (7.4)	66.7 (6.0)	0.6	0.52
BIS-11 attentional impulsiveness score	17.9 (3.0)	17.7 (2.4)	0.4	0.72
BIS-11 motor impulsiveness score	25.0 (3.2)	25.3 (2.3)	−0.5	0.61
BIS-11 non-planning impulsiveness score	13.2 (2.6)	12.3 (1.5)	1.9	0.06
**Clinical characteristics**				
Age at illness onset	23.1 (6.0)	–		
Illness duration in months	17.2 (11.6)	–		
Past depressive episode, *n* (%)	13 (33.3)	–		
YMRS score	1.4 (1.9)	–		
HAM-D score	2.1 (2.6)	–		
PANSS positive symptom score^d^	6.8 (1.5)	–		
PANSS disorganization score^d^	7.2 (0.7)	–		
PANSS negative symptom score^d^	8.9 (2.1)	–		
**Treatment characteristics**				
Antidepressants, *n* (%)	2 (5.1)	–		
Lithium, *n* (%)	9 (23.1)	–		
Sodium Valproate, *n* (%)	18 (46.2)	–		
Antipsychotics^e^, *n* (%)	30 (76.9)	–		
Chlorpromazine equivalents,^f^ mg/day	305.1 (193.1)	–		

*BIS, Barratt Impulsiveness Scale; HAM-D, Hamilton Rating Scale for Depression; PANSS, Positive and Negative Syndrome Scale; YMRS, Young Mania Rating Scale*.

a *Data are presented in mean and standard deviations, except gender and use of medications*.

b *Potential group differences were examined using independent-samples t-tests and chi-square tests for continuous and categorical variables, respectively*.

c *Letter cancellation test performance was measured by the number of omission errors committed, with higher number of omission errors indicating poorer performance*.

d *PANSS positive symptom and disorganization scores were derived on the basis of a previous factor-analytic study on first-episode psychosis patients (36)*.

e *All of the 30 patients who received antipsychotic treatment were on second-generation antipsychotic*.

f *Chlorpromazine equivalents were computed according to (35)*.

### Task Performance and Its Relationships With Clinical and Cognitive Variables

Patients displayed significantly lower adjusted score and explosion rate than controls on the BART (Table 2). Patients also obtained significantly lower cumulative score than controls. We found no significant correlations between BART measures and symptom dimensions in patient sample (Table 3). Risk-taking performance indices were also not correlated with chlorpromazine equivalents in patients who were taking antipsychotic medications at the time of the study. Similarly, no significant correlations were observed between risk-taking performance indices and measures of any cognitive functions (*i.e*., individual cognitive test or cognitive composite scores) or self-reported impulsivity traits in patients and controls (Supplementary Tables 1, 2). Exploratory analyses revealed that patients with antipsychotic treatment did not differ from those without antipsychotic treatment in demographics, cognitive functions, clinical and other treatment characteristics, self-report impulsivity, or any of the three BART performance measures (Supplementary Tables 3, 4). Likewise, our additional subgroup comparison analyses showed lack of significant differences in BART performance measures between patients on *vs*. not on lithium, on*vs*. not on valproate, or with*vs*. without past depressive episode (Supplementary Tables 5-10).

**Table 2:** Comparison of risk-taking performance measures between patients and controls^a^.

**Performance measures^**b**^**	**Patients**	**Controls**	***t***	***p-*value**	**Cohen's *d***
Adjusted score	29.9 (13.5)	41.1 (14.8)	−3.5	0.001	0.79
Rate of exploded balloons	0.3 (0.1)	0.4 (0.1)	−2.8	0.007	0.63
Cumulative score	412.0 (148.1)	518.2 (149.0)	−3.1	0.003	0.71

a *Independent-sample t-tests were performed for patient-control comparisons*.

b *Data are presented in mean and standard deviations*.

**Table 3:** Correlations of risk-taking performance measures with clinical and treatment characteristic in patients^a^.

**Performance measures**	**YMRS**	**HAM-D**	**PANSS POS**	**PANSS DISORG**	**PANSS NEG**	**APD^**b**^**
Adjusted score	0.13	0.21	0.01	−0.10	−0.01	−0.05
Explosion rate	−0.14	0.10	0.03	−0.07	−0.08	−0.01
Cumulative score	0.25	0.27	−0.01	0.14	−0.01	−0.12

*APD, antipsychotic dose; DISORG, Disorganization score; HAM-D, Hamilton Rating Scale for Depression; NEG, Negative symptom score; PANSS, Positive and Negative Syndrome Scale; POS, Positive symptom score; YMRS, Young Mania Rating Scale*.

a *Pearson correlation analyses were performed and r values were presented*.

b *Antipsychotic dose was quantified using chlorpromazine equivalents (mg/daily)*.

## Discussion

To our knowledge, the current study is the first to examine risk-taking behavior among bipolar disorder patients in their initial few years following first-episode mania with psychosis using the BART paradigm. Our results showed that patients made significantly fewer average balloon pumps in unexploded trials and had lower explosion rate than controls, indicating their increased tendency for risk aversion. This is, however, contrary to a recent meta-analysis which found no significant impairment in risk-taking behavior in bipolar disorder, in terms of the pooled estimate for the entire sample or individual behavioral tasks (2). Some previous studies (7, 9, 10) and stratified analysis of the meta-analytic review (2) further revealed elevated risk-taking behavior in bipolar disorder patients, particularly those with bipolar I disorder. Of note, the conservative strategy observed in our study was found to be suboptimal in the context of the BART, as evidenced by lower cumulative scores attained by patients as compared to controls.

Several possible reasons might explain the discrepancy between our findings and those of past studies on the BART performance. First, all bipolar disorder patients recruited in our study were previously presented with psychotic features (in their first-episode mania), whereas samples of prior reports mainly comprised bipolar disorder without psychosis (BD-NP) with varying but small proportion of patients with a history of psychosis (2). Intriguingly, our results were similar to earlier studies on patients with chronic schizophrenia (4, 8, 19, 37) and early non-affective psychosis (20) who also displayed elevated risk aversion in the BART, relative to controls. Thus, it is possible that pathophysiology underlying psychosis manifestation in schizophrenia and bipolar disorder might contribute to the behavioral commonalities on risk-taking propensity. In fact, recent evidence has indicated elevated striatal dopamine synthesis capacity in BD-P, with its magnitude comparable to that of schizophrenia (38). And, dopamine neurotransmission and frontostrital system are both critically involved in decision process under risk (39). Alternatively, a recent study revealed that, patients with schizophrenia and BD-P were significantly and similarly impaired in probabilistic reward-driven learning, but had preserved punishment-driven learning, relative to those with BD-NP (40). Many (41–43), though not all (44, 45), previous studies also found that BD-P was associated with greater cognitive impairment as well as worse clinical and functional outcomes than BD-NP, suggesting that BD-P may be more similar to schizophrenia than to BD-NP. Future research directly comparing BD-P and BD-NP patients on BART performance with adequate sample size would help clarify the potential role of psychosis in bipolar disorder on risk-taking propensity. Second, a large proportion of our patients were treated with antipsychotic medication which may, however, be related to increased risk aversion. An earlier study demonstrated that bipolar disorder patients on antipsychotic behaved significantly more conservative than those without antipsychotic treatment in the BART (8). Notably, our exploratory analysis demonstrated lack of significant difference between patients with and without antipsychotic treatment in any of the BART performance indices, and antipsychotic dose was not correlated with risk-taking measures. Notwithstanding, our results of no significant relationship between antipsychotic status and risk-taking behavior should be treated with caution due to small sample size of our patient subgroup without antipsychotic treatment. Similarly, a larger patient sample size would be required to verify our preliminary findings of lack of significant associations of risk-taking measures with history of prior depressive episode as well as lithium and valproate treatment. Third, heightened risk-taking may evolve over time, depending on illness chronicity and recurrence of mood episodes. Prospective investigation with longer follow-up is required to elucidate the longitudinal trajectory of risk-taking propensity over the course of illness.

Our results that patients did not differ from controls in BIS-11 scores are at odds with most previous studies (14), albeit primarily based on chronically-ill samples, which demonstrated that bipolar disorder was associated with increased self-reported trait impulsivity, even during euthymic state. On the other hand, there is evidence indicating that greater self-reported impulsivity is related to longer illness duration and more frequent mood episodes in bipolar disorder patients (46). It is thus suggested that elevated self-reported impulsivity might partly be attributable to the consequence of illness. In particular, patients with chronic unstable illness course may be more inclined to perceiving impulsivity as a pervasive characteristic, resulting in inflated self-reported scores (8). Conversely, as our patients were in the relatively early illness stage with stable euthymic state following remission from first manic episode, they may thus be less likely to self-perceive impulsivity as a stable trait after illness onset.

We observed no significant correlations between risk-taking measures and symptom variables, self-reported impulsivity and cognitive test performance. Our results thus suggest that altered risk-taking behavior in patients with early-stage bipolar disorder might be relatively independent of specific symptom dimensions and impairment in general cognitive functions. In fact, this is consistent with prior studies which examined the BART in bipolar disorder and revealed absence of any meaningful associations of risk-taking performance with clinical profiles or cognitive functions (6–8). Our findings also concur with the literature consistently showing that risk-taking behavioral indices are not related to (or at best weakly correlated with) self-reported impulsivity in bipolar disorder (2), people with other psychiatric disorders (47), and healthy populations (48). The poor concordance between these *two* measures might partly be explained by the fact that risk-taking and impulsivity in fact represent *two* separable, albeit related, constructs (7, 16). An inherent difference between objective cognitive / performance-based assessment conducted in laboratory settings and subjective evaluation of self-perceived deficits emerged in unstructured real-world environment may also contribute to such lack of concurrence (49). On the other hand, as we focused on clinically-stable, euthymic bipolar disorder patients who had mild-to-minimal symptom levels and comparable BIS-11 scores to controls, the variance in symptom severity and impulsivity ratings might be too small for subtle yet significant associations with risk-taking measures to be detected.

The study results should be interpreted in light of the following methodological limitations. First, our sample size was modest which may compromise the statistical power to detect subtler group difference on risk-taking measures and self-reported impulsivity, particularly the comparison between patients with and without antipsychotic treatment. Second, an absence of a BD-NP subgroup precludes us from clarifying whether the observed altered risk-taking behavior is specifically related to BD-P status or manifested in the early stage of illness in general, irrespective of a history of psychosis. Third, several factors that may potentially contribute to impaired risk-taking behavior such as value representation and attentional bias were not examined in this study. Fourth, the majority of our patients were receiving antipsychotic medication at the time of assessment. Although our analyses revealed no correlations between risk-taking measures and antipsychotic dose, we cannot rule out an effect of dopamine D2 antagonism on decision-making under risk. Fifth, data on the number and clinical profiles of patients who were approached for but declined study inclusion were not available, thereby precluding us from assessing potential participation (selection) bias. Lastly, literature has suggested that risk-taking is a multifaceted construct (48) and thus may not be adequately captured by using a single behavioral paradigm of BART. Future research may consider employing a range of measures to enable a more comprehensive evaluation of risk-taking propensity in bipolar disorder.

## Conclusion

In conclusion, our results indicate that euthymic patients remitted within 3 years from first-episode mania with psychosis exhibit a tendency for risk aversion in the BART paradigm. This observation, albeit preliminary, may be of clinical significance as excessive risk avoidance would result in diminished pursuit of opportunities and poorer functional outcome. Owing to the paucity of existing data, further research is warranted to verify the risk-taking behavioral pattern and its relationship with psychosis and antipsychotic treatment in the early course of bipolar disorder.

## Data Availability Statement

The raw data supporting the conclusions of this article will be made available by the authors, without undue reservation.

## Ethics Statement

The studies involving human participants were reviewed and approved by Institutional Review Board of the University of Hong Kong / Hospital Authority Hong Kong West Cluster. Written informed consent to participate in this study was provided by the participants' legal guardian/next of kin.

## Author Contributions

WC conceptualized, designed the study, revised, and finalized the manuscript. SW and MN conducted data analysis. WC, SW, and MN interpreted the data. SW drafted the manuscript. All authors provided critical feedback to the draft manuscript, reviewed, and approved the final version of the manuscript.

## Funding

This study was supported by the Seed Fund for Basic Research of the University of Hong Kong (201711159052) and the General Research Fund of Research Grants Council (17127417).

## Conflict of Interest

The authors declare that the research was conducted in the absence of any commercial or financial relationships that could be construed as a potential conflict of interest.

## Publisher's Note

All claims expressed in this article are solely those of the authors and do not necessarily represent those of their affiliated organizations, or those of the publisher, the editors and the reviewers. Any product that may be evaluated in this article, or claim that may be made by its manufacturer, is not guaranteed or endorsed by the publisher.
