# Supplementary material for: Altered Risk-Taking Behavior in Early-Stage Bipolar Disorder With a History of Psychosis
Source: Front Psychiatry. 2021 Nov 18;12:763545. doi: 10.3389/fpsyt.2021.763545 (PMC8637446; doi:10.3389/fpsyt.2021.763545)
Supplement: Supplementary file 1 [file Data_Sheet_1.docx]

**Supplementary Materials**

1. **Table S1.** Correlations of risk-taking performance measures with cognitive functions and impulsivity traits in patients.
2. **Table S2.** Correlations of risk-taking performance measures with cognitive functions and impulsivity traits in controls.
3. **Table S3.** Demographics, cognitive functions and clinical characteristics of patients with and without antipsychotic treatment.
4. **Table S4.** Comparison of risk-taking performance measures between patients with and without antipsychotic treatment.
5. **Table S5**. Demographics, cognitive functions and clinical characteristics of patients with and without lithium treatment.
6. **Table S6**. Comparison of risk-taking performance measures between patients with and without lithium treatment.
7. **Table S7**. Demographics, cognitive functions and clinical characteristics of patients with and without valproate treatment.
8. **Table S8**. Comparison of risk-taking performance measures between patients with and without valproate treatment.
9. **Table S9**. Demographics, cognitive functions and clinical characteristics of patients with and without past depressive episode.
10. **Table S10**. Comparison of risk-taking performance measures between patients with and without past depressive episode.

**Table S1** Correlations of risk-taking performance measures with cognitive functions and impulsivity traits in patients.^a^

| Performance measures | Letter number span | Digit symbol | Logical memory | Letter cancellation | General cognition^b^ | BIS-11  total | BIS-11 Attention | BIS-11 Motor | BIS-11 Non-planning |
| --- | --- | --- | --- | --- | --- | --- | --- | --- | --- |
| Adjusted score | 0.08 | 0.03 | 0.20 | -0.13 | 0.269 | 0.05 | 0.12 | -0.12 | 0.06 |
| Explosion rate | 0.03 | 0.03 | 0.09 | -0.21 | 0.078 | 0.03 | 0.03 | -0.07 | 0.11 |
| Cumulative score | 0.17 | 0.17 | 0.22 | -0.10 | 0.315 | -0.01 | 0.04 | -0.11 | -0.02 |

BIS, Barrett Impulsiveness Scale.

^a^ Pearson correlation analyses were performed and r values were presented.

^b^ General cognitive function (i.e., cognitive composite score) of each participant was calculated by averaging the standardized z-scores of individual cognitive tests, with z-score of each cognitive test being computed on the basis of healthy controls’ performance.

**p*<0.05.

**Table S2** Correlations of risk-taking performance measures with cognitive functions and impulsivity traits in controls.^a^

| Performance measures | Letter number span | Digit symbol | Logical memory | Letter cancellation | General cognition^b^ | BIS-11  total | BIS-11 Attention | BIS-11 Motor | BIS-11 Non-planning |
| --- | --- | --- | --- | --- | --- | --- | --- | --- | --- |
| Adjusted score | -0.11 | -0.21 | 0.01 | -0.14 | -0.215 | 0.17 | 0.06 | -0.03 | 0.21 |
| Explosion rate | 0.15 | -0.17 | 0.04 | 0.03 | 0.020 | -0.04 | -0.10 | -0.05 | -0.06 |
| Cumulative score | -0.13 | 0.02 | 0.12 | -0.31 | -0.146 | 0.18 | 0.15 | -0.02 | 0.23 |

BIS, Barrett Impulsiveness Scale.

^a^ Pearson correlation analyses were performed and r values were presented.

^b^ General cognitive function (i.e., cognitive composite score) of each participant was calculated by averaging the standardized z-scores of individual cognitive tests, with z-score of each cognitive test being computed on the basis of healthy controls’ performance.

**p*<0.05.

**Table S3** Demographics, cognitive functions and clinical characteristics of patients with and without antipsychotic treatment.^a^

|  | On antipsychotic^c^ | No antipsychotic | Statistics^b^ |  |
| --- | --- | --- | --- | --- |
| Variables^a^ | (n = 30) | (n = 9) | (t/χ^2^) | *p* value |
| Demographics |  |  |  |  |
| Age in years | 23.9 (6.3) | 26.0 (5.9) | 0.9 | 0.89 |
| Male gender, n (%) | 14 (46.7) | 2 (22.2) | 1.7 | 0.19 |
| Years of education | 13.6 (2.8) | 13.6 (3.1) | -0.01 | 0.99 |
| Cognitive functions |  |  |  |  |
| Letter-number span | 14.5 (4.3) | 13.9 (2.9) | -0.4 | 0.71 |
| Digit symbol coding | 11.6 (3.3) | 12.8 (3.6) | 0.9 | 0.36 |
| Logical memory | 11.0 (4.6) | 8.9 (4.6) | -1.2 | 0.23 |
| Letter cancellation | 3.8 (3.2) | 4.1 (5.2) | 0.2 | 0.81 |
| Impulsivity trait measures |  |  |  |  |
| BIS-11 total score | 67.1 (7.3) | 69.8 (7.8) | 1.0 | 0.34 |
| BIS-11 attentional impulsiveness score | 17.4 (2.8) | 19.7 (3.4) | 2.0 | 0.07 |
| BIS-11 motor impulsiveness score | 24.6 (3.1) | 26.2 (3.4) | 1.4 | 0.18 |
| BIS-11 non-planning impulsiveness score | 13.4 (2.6) | 12.6 (2.3) | -0.8 | 0.41 |
| Clinical characteristics |  |  |  |  |
| Age at illness onset | 22.7 (6.2) | 24.2 (5.5) | 0.6 | 0.52 |
| Past depressive episode, n (%) | 9 (30.0) | 4 (44.4) | 0.6 | 0.43 |
| YMRS score | 1.2 (1.8) | 2.2 (2.1) | 1.5 | 0.15 |
| HAM-D score | 2.3 (2.8) | 1.4 (1.7) | -0.9 | 0.34 |
| PANSS positive symptom score | 6.7 (1.4) | 7.2 (1.7) | 0.9 | 0.37 |
| PANSS disorganization score | 7.2 (0.6) | 7.3 (1.0) | 0.5 | 0.63 |
| PANSS negative symptom score | 9.1 (2.3) | 8.0 (1.0) | -1.5 | 0.15 |
| Treatment characteristics |  |  |  |  |
| Antidepressants, n (%) | 2 (6.7) | 0 | 0.6 | 0.43 |
| Lithium, n (%) | 6 (20.0) | 2 (22.2) | 8.0 | 0.16 |
| Sodium Valproate, n (%) | 13 (43.3) | 5 (55.6) | 7.2 | 0.21 |

BIS Barratt Impulsiveness Scale; HAM-D Hamilton Rating Scale for Depression; PANSS Positive and Negative Syndrome Scale; YMRS Young Mania Rating Scale.

^a^ Data are presented in mean and standard deviations, except gender and use of medications.

^b^ Potential group differences were examined using independent-samples t tests and chi-square tests for continuous

and categorical variables, respectively.

^c^ All of the 30 patients who received antipsychotic treatment were on second-generation antipsychotic.

**Table S4** Comparison of risk-taking performance measures between patients with and without antipsychotic

treatment.^a^

| Performance measures^b^ | On antipsychotic | No antipsychotic | *t* | *p* value | Cohen’s *d* |
| --- | --- | --- | --- | --- | --- |
| Adjusted score | 30.4 (13.3) | 28.0 (14.7) | -0.5 | 0.64 | 0.17 |
| Rate of exploded balloons | 0.33 (0.1) | 0.26 (0.2) | -1.2 | 0.24 | 0.44 |
| Cumulative score | 411.0 (143.0) | 415.3 (174.1) | 0.1 | 0.94 | 0.03 |

^a^ Independent-sample t-tests were performed for patient-control comparisons.

^b^ Data are presented in mean and standard deviations.

**Table S5** Demographics, cognitive functions and clinical characteristics of patients with and without lithium treatment.^a^

|  | On lithium | No lithium | Statistics^b^ |  |
| --- | --- | --- | --- | --- |
| Variables^a^ | (n = 8) | (n = 30) | (t/χ^2^) | *p* value |
| Demographics |  |  |  |  |
| Age in years | 21.4 (2.6) | 25.2 (6.6) | -2.6 | 0.02 |
| Male gender, n (%) | 4 (50.0) | 12 (38.7) | 0.3 | 0.57 |
| Years of education | 13.2 (2.6) | 13.7 (2.9) | -0.4 | 0.70 |
| Cognitive functions |  |  |  |  |
| Letter-number span | 12.4 (2.8) | 14.8 (4.1) | -1.6 | 0.12 |
| Digit symbol coding | 8.8 (2.4) | 12.7 (3.1) | -3.3 | <0.001 |
| Logical memory | 8.5 (3.6) | 11.1 (4.6) | -1.5 | 0.15 |
| Letter cancellation | 5.3 (5.5) | 3.5 (3.1) | 1.2 | 0.23 |
| Impulsivity trait measures |  |  |  |  |
| BIS-11 total score | 70.9 (7.7) | 66.9 (7.2) | 1.4 | 0.18 |
| BIS-11 attentional impulsiveness score | 18.8 (3.2) | 17.7 (3) | 0.8 | 0.41 |
| BIS-11 motor impulsiveness score | 25.8 (4.3) | 24.8 (2.9) | 0.8 | 0.45 |
| BIS-11 non-planning impulsiveness score | 14.5 (1.4) | 12.8 (2.7) | 1.7 | 0.10 |
| Clinical characteristics |  |  |  |  |
| Age at illness onset | 20.6 (3.2) | 23.7 (6.4) | -1.3 | 0.20 |
| Past depressive episode, n (%) | 4 (50.0) | 9 (29.0) | 1.2 | 0.27 |
| YMRS score | 1.5 (1.7) | 1.4 (1.9) | 0.1 | 0.92 |
| HAM-D score | 2.8 (2.2) | 1.9 (2.7) | 0.8 | 0.43 |
| PANSS positive symptom score | 7.5 (1.9) | 6.7 (1.4) | 1.5 | 0.15 |
| PANSS disorganization score | 7.3 (0.7) | 7.2 (0.7) | 0.1 | 0.93 |
| PANSS negative symptom score | 10.4 (3.9) | 8.5 (1.1) | 1.4 | 0.22 |
| Treatment characteristics |  |  |  |  |
| Antipsychotics, n (%) | 6 (75.0) | 24 (77.4) | 0.1 | 0.89 |
| Antidepressants, n (%) | 0 (0.0) | 2 (6.5) | 0.9 | 0.33 |
| Sodium Valproate, n (%) | 2 (2.5) | 16 (51.6) | 1.9 | 0.17 |

BIS Barratt Impulsiveness Scale; HAM-D Hamilton Rating Scale for Depression; PANSS Positive and Negative Syndrome Scale; YMRS Young Mania Rating Scale.

^a^ Data are presented in mean and standard deviations, except gender and use of medications.

^b^ Potential group differences were examined using independent-samples t tests and chi-square tests for continuous

and categorical variables, respectively.

**Table S6** Comparison of risk-taking performance measures between patients with and without lithium

treatment.^a^

| Performance measures^b^ | On lithium | No lithium | *t* | *p* value | Cohen’s *d* |
| --- | --- | --- | --- | --- | --- |
| Adjusted score | 29.3 (9.2) | 30.0 (14.5) | -0.1 | 0.90 | 0.06 |
| Rate of exploded balloons | 0.3 (0.1) | 0.3 (0.2) | -0.6 | 0.56 | 0.24 |
| Cumulative score | 435.1 (109.4) | 406.0 (157.8) | 0.5 | 0.63 | 0.21 |

^a^ Independent-sample t-tests were performed for patient-control comparisons.

^b^ Data are presented in mean and standard deviations.

**Table S7** Demographics, cognitive functions and clinical characteristics of patients with and without valproate treatment.^a^

|  | On valproate | No valproate | Statistics^b^ |  |
| --- | --- | --- | --- | --- |
| Variables^a^ | (n = 18) | (n = 21) | (t/χ^2^) | *p* value |
| Demographics |  |  |  |  |
| Age in years | 25.8 (7.2) | 23.2 (4.9) | 1.3 | 0.20 |
| Male gender, n (%) | 7 (38.9) | 9 (42.9) | 0.1 | 0.80 |
| Years of education | 14.3 (2.3) | 13 (3.1) | 1.4 | 0.16 |
| Cognitive functions |  |  |  |  |
| Letter-number span | 14.6 (4.4) | 14.1 (3.7) | 0.3 | 0.75 |
| Digit symbol coding | 12.3 (3.7) | 11.5 (3.1) | 0.7 | 0.49 |
| Logical memory | 11.2 (4.4) | 10 (4.6) | 0.8 | 0.44 |
| Letter cancellation | 3.1 (2.2) | 4.5 (4.5) | -1.3 | 0.20 |
| Impulsivity trait measures |  |  |  |  |
| BIS-11 total score | 67.2 (6.3) | 68.1 (8.3) | -0.4 | 0.69 |
| BIS-11 attentional impulsiveness score | 17.8 (2.5) | 18 (3.5) | -0.2 | 0.83 |
| BIS-11 motor impulsiveness score | 24.9 (3.1) | 25 (3.3) | -0.1 | 0.96 |
| BIS-11 non-planning impulsiveness score | 13.2 (2.5) | 13.2 (2.7) | -0.1 | 0.98 |
| Clinical characteristics |  |  |  |  |
| Age at illness onset | 24.3 (7.1) | 22 (4.8) | 1.2 | 0.23 |
| Past depressive episode, n (%) | 7 (38.9) | 6 (28.6) | 0.5 | 0.50 |
| YMRS score | 1.5 (1.9) | 1.4 (1.9) | 0.20 | 0.85 |
| HAM-D score | 1.3 (1.6) | 2.8 (3.1) | -2.0 | 0.05 |
| PANSS positive symptom score | 6.3 (0.8) | 7.2 (1.8) | -2.1 | 0.05 |
| PANSS disorganization score | 7.2 (0.7) | 7.3 (0.7) | -0.5 | 0.61 |
| PANSS negative symptom score | 8.6 (1) | 9.1 (2.7) | -0.7 | 0.48 |
| Treatment characteristics |  |  |  |  |
| Antipsychotics, n (%) | 13 (72.2) | 17 (81.0) | 0.4 | 0.52 |
| Antidepressants, n (%) | 1 (5.6) | 1 (4.8) | 0.1 | 0.91 |
| Lithium, n (%) | 2 (11.1) | 6 (28.6) | 1.9 | 0.17 |

BIS Barratt Impulsiveness Scale; HAM-D Hamilton Rating Scale for Depression; PANSS Positive and Negative Syndrome Scale; YMRS Young Mania Rating Scale.

^a^ Data are presented in mean and standard deviations, except gender and use of medications.

^b^ Potential group differences were examined using independent-samples t tests and chi-square tests for continuous

and categorical variables, respectively.

**Table S8** Comparison of risk-taking performance measures between patients with and without valproate

treatment.^a^

| Performance measures^b^ | On valproate | No valproate | *t* | *p* value | Cohen’s *d* |
| --- | --- | --- | --- | --- | --- |
| Adjusted score | 27.1 (14.6) | 32.3 (12.3) | -1.2 | 0.24 | 0.38 |
| Rate of exploded balloons | 0.3 (0.2) | 0.3 (0.1) | -1.0 | 0.32 | 0.27 |
| Cumulative score | 383.6 (170.4) | 436.4 (125.5) | -1.1 | 0.27 | 0.35 |

^a^ Independent-sample t-tests were performed for patient-control comparisons.

^b^ Data are presented in mean and standard deviations.

**Table S9** Demographics, cognitive functions and clinical characteristics of patients with and without past depressive episode.^a^

|  | Past depressive episode  (n = 13) | No depressive episode  (n = 26) | Statistics^b^ |  |
| --- | --- | --- | --- | --- |
| Variables^a^ |  |  | (t/χ^2^) | *p* value |
| Demographics |  |  |  |  |
| Age in years | 26.5 (7.5) | 23.4 (5.2) | 1.6 | 0.13 |
| Male gender, n (%) | 3 (23.1) | 13 (50.0) | 2.6 | 0.11 |
| Years of education | 14.2 (3) | 13.3 (2.7) | 0.9 | 0.35 |
| Cognitive functions |  |  |  |  |
| Letter-number span | 13.5 (3.4) | 14.7 (4.2) | -0.9 | 0.38 |
| Digit symbol coding | 10.7 (3.1) | 12.5 (3.3) | -1.6 | 0.12 |
| Logical memory | 9.9 (4.3) | 10.9 (4.6) | -0.6 | 0.52 |
| Letter cancellation | 3.4 (4.4) | 4.1 (3.4) | -0.5 | 0.59 |
| Impulsivity trait measures |  |  |  |  |
| BIS-11 total score | 70.2 (7.1) | 66.5 (7.4) | 1.5 | 0.15 |
| BIS-11 attentional impulsiveness score | 18.2 (2.9) | 17.8 (3.1) | 0.4 | 0.69 |
| BIS-11 motor impulsiveness score | 26.5 (3.5) | 24.2 (2.8) | 2.3 | 0.03 |
| BIS-11 non-planning impulsiveness score | 14 (2.4) | 12.8 (2.6) | 1.4 | 0.16 |
| Clinical characteristics |  |  |  |  |
| Age at illness onset | 25.4 (7.4) | 21.9 (5) | 1.7 | 0.09 |
| YMRS score | 1.7 (2.3) | 1.3 (1.6) | 0.6 | 0.55 |
| HAM-D score | 2.1 (2) | 2.1 (2.9) | -0.1 | 0.97 |
| PANSS positive symptom score | 6.9 (1.5) | 6.8 (1.5) | 0.1 | 0.94 |
| PANSS disorganization score | 7.2 (0.6) | 7.3 (0.8) | -0.5 | 0.64 |
| PANSS negative symptom score | 8.7 (1.1) | 9 (2.4) | -0.4 | 0.71 |
| Treatment characteristics |  |  |  |  |
| Antipsychotics, n (%) | 9 (69.2) | 21 (80.8) | 0.6 | 0.43 |
| Antidepressants, n (%) | 1 (7.7) | 1 (3.8) | 0.2 | 0.62 |
| Lithium, n (%) | 4 (30.8) | 4 (15.4) | 1.2 | 0.27 |
| Sodium valproate, n (%) | 7 (53.8) | 11 (42.3) | 0.5 | 0.50 |

BIS Barratt Impulsiveness Scale; HAM-D Hamilton Rating Scale for Depression; PANSS Positive and Negative Syndrome Scale; YMRS Young Mania Rating Scale.

^a^ Data are presented in mean and standard deviations, except gender and use of medications.

^b^ Potential group differences were examined using independent-samples t tests and chi-square tests for continuous

and categorical variables, respectively.

**Table S10** Comparison of risk-taking performance measures between patients with and without past depressive

episode.^a^

| Performance measures^b^ | Past depressive episode | No depressive episode | *t* | *p* value | Cohen’s *d* |
| --- | --- | --- | --- | --- | --- |
| Adjusted score | 26.0 (9.9) | 31.8 (14.8) | -1.3 | 0.21 | 0.46 |
| Rate of exploded balloons | 0.3 (0.1) | 0.3 (0.2) | -1.1 | 0.30 | 0.38 |
| Cumulative score | 399.8 (147.4) | 418.1 (151.3) | -0.4 | 0.72 | 0.12 |

^a^ Independent-sample t-tests were performed for patient-control comparisons.

^b^ Data are presented in mean and standard deviations.
